# Purple Sweet Potato Ameliorates High-Fat Diet-Induced Visceral Adiposity by Attenuating Inflammation and Promoting Adipocyte Browning

**DOI:** 10.1021/acs.jafc.4c08799

**Published:** 2025-02-03

**Authors:** Chi-Hua Yen, Ming-Hui Chiang, Yi-Chen Lee, Erl-Shyh Kao, Huei-Jane Lee

**Affiliations:** 1 Department of Family and Community Medicine, Chung Shan Medical University Hospital, No. 110, Sec. 1, Jianguo N. Road, South Dist., Taichung City 40221, Taiwan; 2 School of Medicine, Chung Shan Medical University, No. 110, Sec. 1, Jianguo N. Road, South Dist., Taichung City 40221, Taiwan; 3 Institute of Medicine, Chung Shan Medical University, No. 110, Sec. 1, Jianguo N. Road, South Dist., Taichung City 40221, Taiwan; 4 Department of Nutrition Therapy, E-Da Cancer Hospital, No.21, Yida Road, Jiao-su Village, Yan-chao District, Kaohsiung City 82400, Taiwan; 5 Department of Nutrition Therapy, E-Da Hospital, No.1, Yida Road, Jiao-su Village, Yan-chao District, Kaohsiung City 82400, Taiwan; 6 Department of Nutrition, College of Medicine, I-Shou University, No.1, Sec. 1, Syuecheng Rd., Dashu District, Kaohsiung City 84001, Taiwan; 7 Department of Beauty Science, National Taichung University of Science and Technology, No. 193, Section 1, Sanmin Road, West District, Taichung City 40302, Taiwan; 8 Department of Biochemistry, School of Medicine, Chung Shan Medical University, Taichung 40221, Taiwan. No. 110, Sec. 1, Jianguo N. Road, South Dist., Taichung City 40221, Taiwan; 9 Department of Clinical Laboratory, Chung Shan Medical University Hospital, Taichung 40201, Taiwan

**Keywords:** high-fat diet, adiposity, visceral fat, inflammasome, adipocyte browning, purple sweet potato

## Abstract

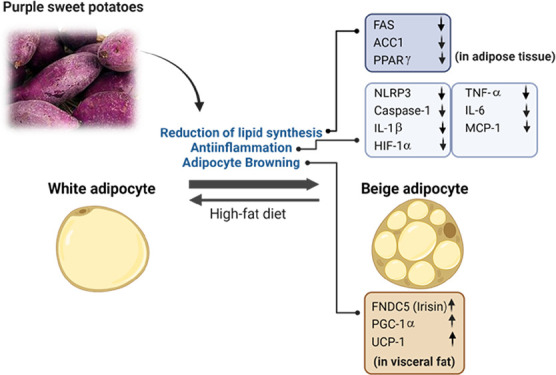

Accumulation of visceral fat has been reported to increase systemic inflammation. Purple sweet potato (*Ipomoea batatas* L., PSP), known for its anthocyanin content, potentiates in mitigating oxidative stress. This study aimed to investigate the underlying mechanisms by which PSP influences body fat deposition. Five-week-old male Sprague–Dawley rats (*n* = 5) were fed a 43% high-fat diet (HFD) for 2 weeks to induce obesity, followed by 19 weeks of HFD supplemented with 5% PSP. PSP significantly improved body weight and reduced visceral fat mass and adipocyte size. In visceral and subcutaneous adipose tissues, PSP significantly downregulated proteins of FAS, ACC1, and PPARγ with inflammatory markers TNF-α, IL-6, and MCP-1. PSP reduced the proteins of inflammasome components, NLRP3, caspase-1, IL-1β, and HIF-1α. PSP increased the proteins associated with adipose tissue browning, FNDC5, PGC-1α, and UCP-1, particularly in visceral adipose tissue. In conclusion, PSP effectively reduced visceral fat accumulation, attenuated inflammation, and promoted adipocyte browning.

## 1 Introduction

Obesity and its associated metabolic disorders have prompted extensive research into the underlying mechanisms of fat accumulation and its impact on systemic inflammation and chronic diseases.^1^ The accumulation of visceral fat has been recognized as a major contributor to metabolic dysfunction. An excess fat tissue alters the secretion of adipokines and proinflammatory cytokines, such as tumor necrosis factor-alpha (TNF-α), interleukin-6 (IL-6), and monocyte chemoattractant protein-1 (MCP-1), which are closely linked to the development of insulin resistance, type 2 diabetes, and cardiovascular diseases.^2^

Peroxisome proliferator-activated receptor gamma (PPARγ), sterol-regulatory element binding protein-1, stearoyl-CoA desaturase (SCD-1), acetyl-CoA carboxylase 1 (ACC1), and fatty acid synthase (FAS) are key regulators of fatty acid synthesis.^2^ Lipid accumulation impairs the regulation of the molecules orchestrated in lipid synthesis. Previous studies have shown that enhanced PPARγ expression leads to lipid accumulation in the liver of patients with nonalcoholic fatty liver disease.^2,3^ In liver and adipose tissue, increasing ACC1 results in triglyceride accumulation.^4,5^ FAS in adipocytes is associated with obesity-induced insulin resistance, steatosis of liver and adipose tissue inflammation with the secretion of pro-inflammatory cytokines such as IL-1α, IL-1β, IL-6, IL-8, and MCP-1.^6,7^

The nucleotide-binding oligomerization domain leucine-rich repeat and pyrin domain containing 3 (NLRP3) inflammasome is a critical component in the innate immune system that mediates caspase-1 activation and the secretion of the proinflammatory cytokines IL-1β or IL-18 in response to infection and cellular damage. A report of clinical trial indicates that abnormal activation of the NLRP3 inflammasome is associated with inflammatory diseases such as Alzheimer’s disease, type 2 diabetes, and atherosclerosis.^8^ Excessive fat activates the NLRP3 inflammasome to increase IL-1β and lead to metabolic diseases.^9^ Studies indicate that feeding mice a HFD increases lipogenesis and triglyceride production, with an increase of IL-1β that plays a critical role in hepatic steatosis. It has been confirmed that obese patients have significantly elevated serum levels of IL-1β in clinical studies.^10,11^

Adipose tissue browning, characterized by the conversion of white adipocytes to beige adipocytes, is associated with enhanced thermogenesis and energy expenditure, making it a potential therapeutic target for obesity management.^12^ Fibronectin type III domain containing 5 (FNDC5) is a membrane protein that is cleaved to secrete irisin from the C-terminal. Irisin expression is regulated by peroxisome proliferator-activated receptor gamma coactivator (PGC)-1α. PGC-1α regulates uncoupling protein (UCP)-1 expression to promote thermogenesis in brown adipose tissue.^13−15^ UCP-1 expression is lower in white adipose tissue, which possess the ability to serve as a possible target for obesity treatment.^16^ Previous studies have shown that brown adipose tissue positively affects metabolism and energy balance in mice. The activation of UCP-1 triggers the browning of white adipose tissue into beige adipose tissue and reduces fat accumulation.^16^

In recent years, dietary interventions have gained attention as a potential means of mitigating obesity and its associated metabolic complications.^17−20^ Among the various functional foods studied, purple sweet potato (*Ipomoea batatas* L., PSP) has emerged as a promising candidate due to its anthocyanin content.^21,22^ Anthocyanins, a class of polyphenolic compounds, are known for their antioxidant, anti-inflammatory, and lipid-modulating properties. PSP was fermented using Lactobacillus and subsequently extracted with alcohol.^23^ The resulting PSP extract was dissolved in dimethyl sulfoxide and administered to animal models and cell cultures. This study demonstrated that PSP supplementation significantly reduced body weight, serum cholesterol levels, and adipocyte size in high-fat diet (HFD)-induced obese animal models. Furthermore, PSP supplementation modulated lipid metabolism in adipose tissue and alleviated oxidative stress in vitro.^23^ However, while the beneficial effects of PSP on oxidative stress and lipid homeostasis are documented, the role of PSP in regulating body fat distribution, particularly visceral fat, remains unclear. Moreover, organic solvent-extracted PSP ferment is not a commonly consumed dietary component. It is ideal to investigate the effects and underlying mechanisms of a more bioavailable PSP formulation on body fat regulation.

In this study, dried PSP powder was administered to HFD-fed animal models. To investigate the potential mechanisms by which PSP may modulate adipose tissue inflammation and browning, this study examined the impact of PSP supplementation on visceral fat accumulation and related molecular pathways in HFD-induced obese rats. By examining the expression of key proteins involved in lipid metabolism, inflammation, and adipose tissue browning, the underlying mechanisms were proposed through which PSP modulates body fat and inflammatory responses in adipose tissue. The findings could provide new insights into the potential therapeutic applications of PSP in the prevention and management of obesity and its related metabolic disorders.

## 2 Materials and Methods

### 2.1 PSP Preparation

PSP powder was generously provided by Yi-yeh Biotechnology Co. (Taichung, Taiwan). Fresh purple sweet potatoes were peeled, cut into small pieces, shadow-dried, and then mechanically ground into a fine powder. The powder was stored at −20 °C until use. Each 100 g of PSP powder contained 94.1 g of carbohydrates, 2.4 g of protein, and 0.8 g of fat. The anthocyanin content of the PSP powder was analyzed using high-performance liquid chromatography (HPLC) with a Dionex Ultimate 3000 series dual low-pressure ternary gradient pump (Dionex Softron GmbH, Germering, Germany) and an Ultimate 3000 series photodiode array detector. Three anthocyanin peaks, identified as delphinidin-3-O-glucoside, cyanidin-3-O-glucoside, and petunidin-3-O-glucoside, were detected in the chromatogram using diode array detection at 530 nm. Quantification of anthocyanins was performed by comparing the HPLC retention times with those of standard compounds, revealing that 100 g of PSP powder contained approximately 8.4 mg of anthocyanins.

### 2.2 Animals

Male Sprague–Dawley rats (BioLASCO Taiwan Co., Ltd.), aged 5 weeks, were housed under controlled conditions with a temperature of 25 °C, 50%–60% humidity, and a 12-h light-dark cycle. The experimental protocol was approved by the Institutional Animal Care and Use Committee of Chung Shan Medical University (IACUC: 2405). After a one-week acclimatization period, 5 rats were separated as a control group on a normal diet (AIN-93 M, Bio-Serv, Flemington, NJ, USA, 3.58 kcal/g, 4.1% calories from fat, n = 5), while the remaining 15 rats were fed a high-fat diet (HFD) for 2 weeks to induce a significant increase in body weight (362.27 ± 21.52 g) compared to the control group (322.36 ± 14.77 g)(*P* < 0.05)(Table S1). Subsequently, the HFD-fed rats were randomly assigned to three groups (n = 5 per group): HFD group, AIN-93 M diet supplemented with lard, 4.35 kcal/g, 43% calories from fat;^24^ PSP group, HFD mixed with 5% (w/w) purple sweet potato powder, fed every day; S (statin) group, a HFD+atorvastatin group, HFD with atorvastatin, a statin drug known for its lipid-lowering effects, at 10 mg/kg body weight. administered via gavage 3 times per week. Food intake and body weight were recorded daily (Table S1 and 2). After an additional 19 weeks of treatment, the rats following an 8-h fasting period were euthanized using carbon dioxide asphyxiation. Blood, subcutaneous (inguinal) and visceral (epididymal, perirenal, mesenteric) adipose tissues, were collected. The organs including heart, liver, spleen and kidney were weighed (Table S3). A portion of each epididymal adipose tissue was fixed in formalin for histological analysis, while the remaining tissue was stored at −80 °C for subsequent analyses.

### 2.3 Serum Analysis

Blood samples were collected after the sacrifice of the animals. Concentrations of serum triglycerides, total cholesterol, LDL-cholesterol, HDL-cholesterol, and glucose were measured using an automatic clinical chemistry analyzer (Toshiba TBA120 FR, Japan). HFD induction rate = [(value of HFD group–value of control group)/value of control group] x 100%; treatment reduction rate = [(value of treatment group–value of HFD group)/(value of HFD – value of control)] x 100%.

### 2.4 Histological Examination

The inguinal and mesenteric adipose tissues were fixed in 10% buffered formaldehyde, followed by hematoxylin and eosin (H&E) staining for histological analysis. The adipocyte areas of adipose tissues were examined using a microscope (Olympus, Tokyo, Japan; 200× magnification). Ten randomly selected images were analyzed using the ImageXpress PICO imaging system, and adipocyte were defined using TissueFAXS Viewer software. The areas of adipocyte were quantified using ImageJ software (National Institutes of Health, USA) to represent the adipocyte size.

### 2.5 Immunoblot Analysis

Approximately 0.1 g of adipose tissue was homogenized in 1000 μL of radioimmunoprecipitation assay buffer containing 10 μL of protease inhibitors (ab271306, Abcam Ltd., Cambridge, UK) using a homogenizer (Bertin, K0668, France). The homogenate was centrifuged at 12,000 g for 10 min at 4 °C (Model 3700, KUBOTA, Osaka, Japan). The supernatant was collected, frozen at −20 °C to remove lipids, and then centrifuged again under the same conditions to obtain the protein extract. Protein concentrations were determined using the Bio-Rad protein assay with bovine serum albumin as the standard. Thirty μg of protein was separated by sodium dodecyl sulfate-polyacrylamide gel electrophoresis and transferred onto a polyvinylidene difluoride membrane (Millipore, MA, USA). The membrane was blocked with EZblocker (Protein-Free Blocking Buffer; Gene Pure, Taiwan) for 10 min, washed three times with TBST buffer (50 mM Tris, 149 mM NaCl, 0.2% Tween 20) for 10 min each, and incubated with primary antibodies (TNF-α, 1:1000, NBP3–11621; IL-6, 1:1000, NBP2–16957; MCP-1, 1:1000, NBP2–41209; IL-1β, 1:1000, NBP1–42767, Novus Biologicals, Colorado, USA; FAS, 1:1000, GTX109833; NLRP3, 1:1000, GTX1333569; FNDC5, 1:1000, GTX03466, Gene Tex, CA, USA; ACC1, 1:1000, 4190; cleaved Caspase-1, 1:1000, 4199; UCP-1, 1:1000, 72298, Cell Signaling, Massachusetts, USA; PPARγ, 1:1000, SC-7196; HIF-1α, 1:1000, SC-53546, Santa Cruz, CA, USA; PCG-1α, 1:1000, ST1203, Merck, Darmstadt, DE). Following primary antibody incubation, the membrane was treated with antimouse horseradish peroxidase (HRP)-conjugated secondary antibodies (GE Healthcare, Buckinghamshire, UK). Detection was performed using enhanced chemiluminescence (ECL) reagents and visualized on ECL hyperfilm with a UVP ChemStudio Touch imaging system (AnalytikJena, Germany). The calculation formula was shown as the following, HFD induction rate = [(value of HFD group–value of control group)/value of control group] x 100%; treatment reduction rate = [(value of treatment group–value of HFD group)/(value of HFD – value of control)] x 100%.

### 2.6 Statistical Analysis

All experimental data were expressed as mean ± standard deviation (SD). Statistical significance was set at a probability level of *P* < 0.05. Analysis of variance followed by Duncan’s multiple range test was used to evaluate differences between groups (SigmaStat 4.0 and SigmaPlot 10.0, Jandel Scientific Software, Corte Madera, CA, USA). Post hoc analysis was performed using the least significant difference test.

## 3 Results

### 3.1 Effects of PSP on Body Weight, Energy Intake, and Body Fat

After 2 weeks of HFD feeding, the rats were fed HFD supplemented with PSP for 19 weeks. The food intake was significantly lower in the HFD group compared to the control group (*p* < 0.01). However, the energy intake was no significant differences among the groups (Table 1). The rat fed HFD showed a significant 23.6% increase in final body weight compared to the control group (*p* < 0.05), whereas the groups of PSP and S were shown significant 76.5 and 55.1% of reduction in body weight, respectively, compared to HFD group (Table 1). The inguinal, epididymal, mesenteric, and perirenal fat tissues were weighed and divided by body weight to analyze its relative values. As shown in Table 1, the HFD group exhibited significant increases of 72.0% in perirenal, 150.7% in mesenteric, and 87.7% in inguinal adipose tissue compared to the control group. Notably, the relative values were reduced significantly by 68.2% in perirenal and 71.0% in mesenteric adipose tissue, respectively, in the PSP group compared to the HFD group. The relative value was reduced significantly by 48.6% in mesenteric adipose tissue in the S group compared to the HFD group.

**Table 1:** Food Intake, Body Weight, and Adipose Mass in Sprague Dawley Rats Exposed to HFD^a^

	Control	HFD	PSP	S
Food intake (g/day)	27.7 ± 2.2a	20.7 ± 1.3b	20.8 ± 1.7b	20.7 ± 1.6b
Energy intake (kcal/day)	80.2 ± 6.6a	87.1 ± 5.5b	87.3 ± 6.6b	87.7 ± 7.1b
Initial body weight (g)	249.0 ± 1.4a	250.6 ± 2.8a	248.4 ± 1.7a	250.3 ± 0.9a
Body weight after 2-week HFD	322.4 ± 14.8a	362.3 ± 21.5b	361.5 ± 21.7b	362.9 ± 20.5b
Final body weight (g)	555.2 ± 26.4a	686.0 ± 47.0b	585.8 ± 25.9ac	613.8 ± 16.8c
Epididymal adipose^b^	17.1 ± 1.9a	23.6 ± 4.0b	19.6 ± 3.0ab	21.3 ± 3.7ab
Perirenal adipose^b^	23.6 ± 2.8a	40.6 ± 7.9b	29.0 ± 4.0a	32.0 ± 6.1ab
Mesenteric adipose^b^	7.1 ± 1.7a	17.8 ± 6.9b	10.2 ± 1.1c	12.6 ± 1.9a
Inguinal adipose^b^	19.5 ± 3.2a	36.6 ± 9.3b	33.9 ± 4.0b	34.2 ± 5.0b

a The animals were treated with a high fat diet (HFD) for 2 weeks to increase body wight, then fed HFD or supplemented with PSP (5%) or S (10 mg/kg of atorvastatin) for 19 weeks (*n* = 5). The control group was given the control diet. Values (mean ± SD) not sharing a common letter in the same row are significantly different (*p* < 0.05).

b Values were represented as g of adipose tissue/kg of body weight.

### 3.2 Effects of PSP on Serum Lipid and Glucose Levels

Serum lipid levels were elevated in the HFD group, with total cholesterol and LDL-cholesterol levels increasing by 17.7 and 47.8%, respectively. While the PSP-treated group exhibited reductions in these parameters, only the decrease in total cholesterol was statistically significant compared to the HFD group (*p* < 0.05). Serum triglyceride levels were significantly lower in the HFD group relative to the control group; however, no significant differences in triglyceride levels were observed between the PSP and S groups when compared to the HFD group. Blood glucose levels were significantly elevated in the HFD group compared to the control, with no significant differences in glucose levels between the PSP, S, and HFD groups (Table 2).

**Table 2:** Effects of PSP on the Plasma Lipid and Gulcose in Sprague Dawley Rats Exposed to HFD^a^

	Control	HFD	PSP	S^b^
Triglycerides (mg/dL)	111.6 ± 14.6a	72.6 ± 22.3b	66.8 ± 7.6b	79.0 ± 22.5b
Total cholesterol (mg/dL)	57.6 ± 5.5a	67.8 ± 5.1b	57.0 ± 3.1a	53.4 ± 8.5a
LDL-cholesterol (mg/dL)	4.6 ± 0.9a	6.8 ± 1.6b	6.6 ± 0.9b	5.4 ± 1.5ab
HDL-cholesterol (mg/dL)	39.1 ± 4.4a	35.4 ± 4.6a	35.3 ± 1.5a	34.8 ± 6.3a
Glucose (mg/dL)	151.0 ± 26.1a	190.4 ± 9.8b	198.0 ± 6.1b	181.2 ± 26.3ab

a The animals were treated with a high fat diet (HFD) along or supplemented with PSP (5%) or S (10 mg/kg of atorvastatin) for 19 weeks. The control group was given the control diet.

b Values (means ± SD, *n* = 5) not sharing a common letter in the same row are significantly different (*p* < 0.05).

### 3.3 PSP Reduces Subcutaneous and Visceral Adipocyte Size

**Figure 1:**
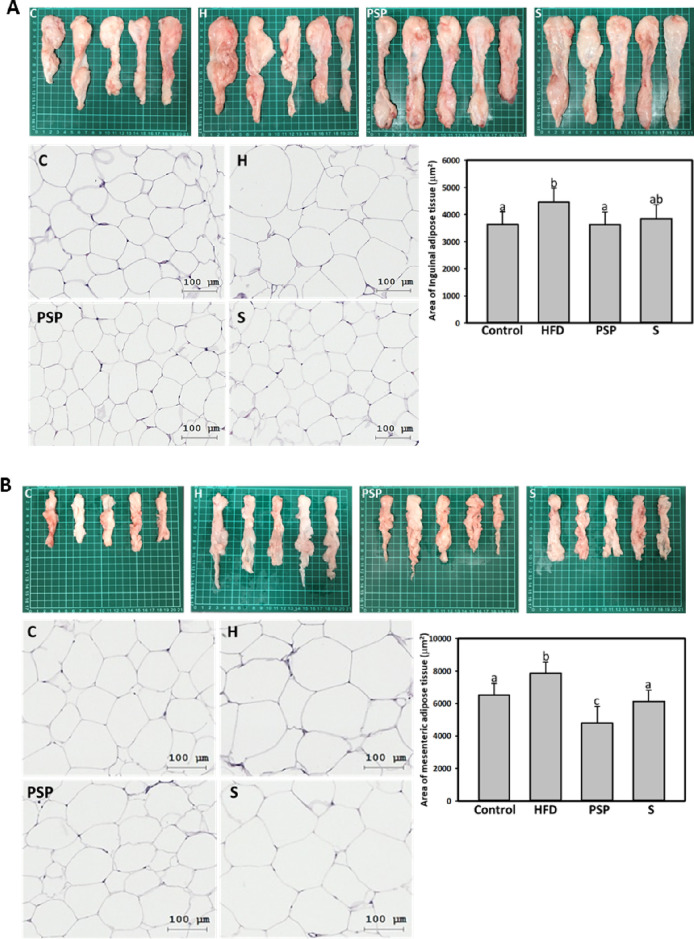
PSP reduced adipocyte size in HFD rats. The HFD-induced obese animals were treated with HFD alone or supplemented with PSP (5%) or S (atorvastatin) for additional 19 weeks. The adipose tissues were obtained to perform histological analysis. The control group was given the control diet. A, subcutaneous (inguinal) and B, visceral (mesenteric) adipose were shown with the histological image (scale bar, 50 μm) and the quantification of adipocytes. In the quantification of adipocyte size, values (mean ± SD) not sharing a common letter in the same row are significantly different (*p* < 0.05).

According to the changes of body fat shown in Table 1, in visceral fat, PSP reduced mesenteric adipose tissue much more than perirenal adipose tissue. To compare the adipocyte size of visceral and subcutaneous fat, histological analysis was performed to measure the adipocyte size in inguinal (subcutaneous) and mesenteric (visceral) adipose tissue. In the results of subcutaneous and visceral adipose tissue, significant increases in adipocyte size by 22.27 and 20.9%, respectively, were shown in the HFD group (*p* < 0.05). In contrast, PSP or S treatment resulted in a significant 102.4 or 75.6% reduction in the subcutaneous adipocyte area (*p* < 0.05) (Figures 1A and B). Analysis of visceral adipocyte size showed that the PSP or S-treated groups showed a reduction of 226.4 or 127.8% (Figures 1A and B). The results revealed that PSP reduces subcutaneous and visceral adipocyte size, interestingly, PSP reduced adipocyte size in visceral adipose tissue more dominantly than that in subcutaneous adipose tissue.

### 3.4 PSP Reduces the Protein Levels of Lipid Synthesis in Adipose Tissue

**Figure 2:**
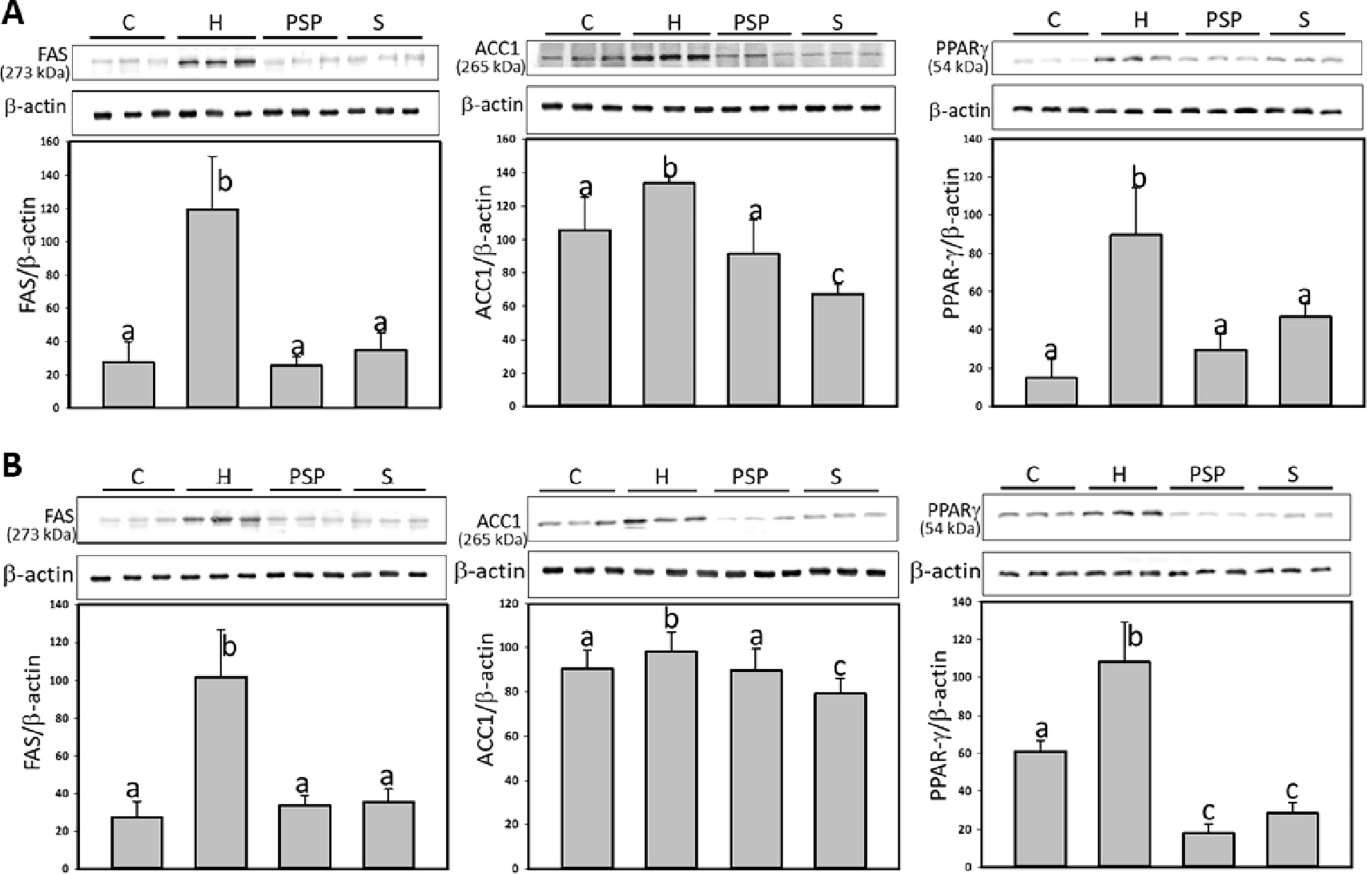
PSP reduced the proteins of lipid synthesis in adipose tissue of HFD rats. Immunoblot examination and ratio values calculated from triplicate experiments were represented. A, subcutaneous (inguinal); B, visceral (mesenteric) adipose. β-actin was used as loading control. The relative image density was quantified by the densitometer. Values (mean ± SD) not sharing a common letter in the same row are significantly different (*p* < 0.05).

To explore the effects of PSP on lipid synthesis, the protein expression levels of FAS, ACC1, and PPARγ were assessed in subcutaneous and visceral fat tissues. As depicted in Figures 2A, HFD led to increased expression levels of FAS, ACC1, and PPARγ in subcutaneous adipose tissue, with significant increases observed for all proteins. PSP treatments significantly reduced the expression levels of FAS, ACC1, and PPARγ by 102.7, 105.6, and 80.4%, respectively; S treatments significantly reduced those by 92.5, 236.5, and 57.0%, respectively, compared to H group. In visceral adipose tissue, significant reductions in the expression of these proteins were observed following PSP treatment, FAS, ACC1, and PPARγ by 91.4, 108.5, and 191.7%, respectively; S treatments significantly reduced FAS, ACC1, and PPARγ by 88.9, 244.0, and 169.6%, respectively, respectively, compared to H group (*p* < 0.05) (Figures 2B).

### 3.5 PSP Inhibits Inflammation-Related Protein Expression in Adipose Tissue

**Figure 3:**
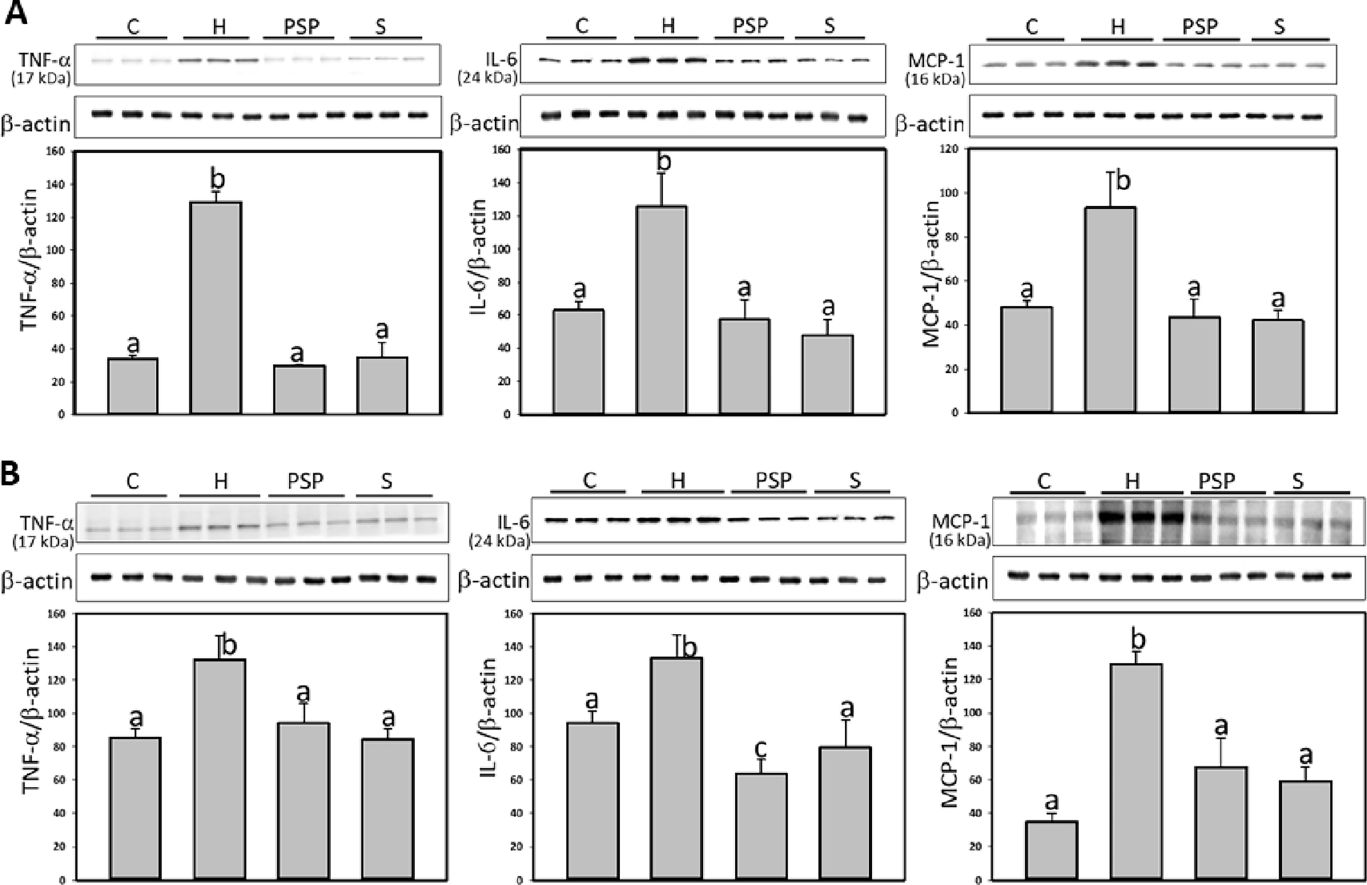
PSP inhibited the level of pro-inflammatory cytokines in adipose tissue of HFD rats. A, subcutaneous (inguinal); B, visceral (mesenteric) adipose. β-actin was used as loading control. The relative image density was quantified by the densitometer. Values (mean ± SD) not sharing a common letter in the same row are significantly different (*p* < 0.05).

**Figure 4:**
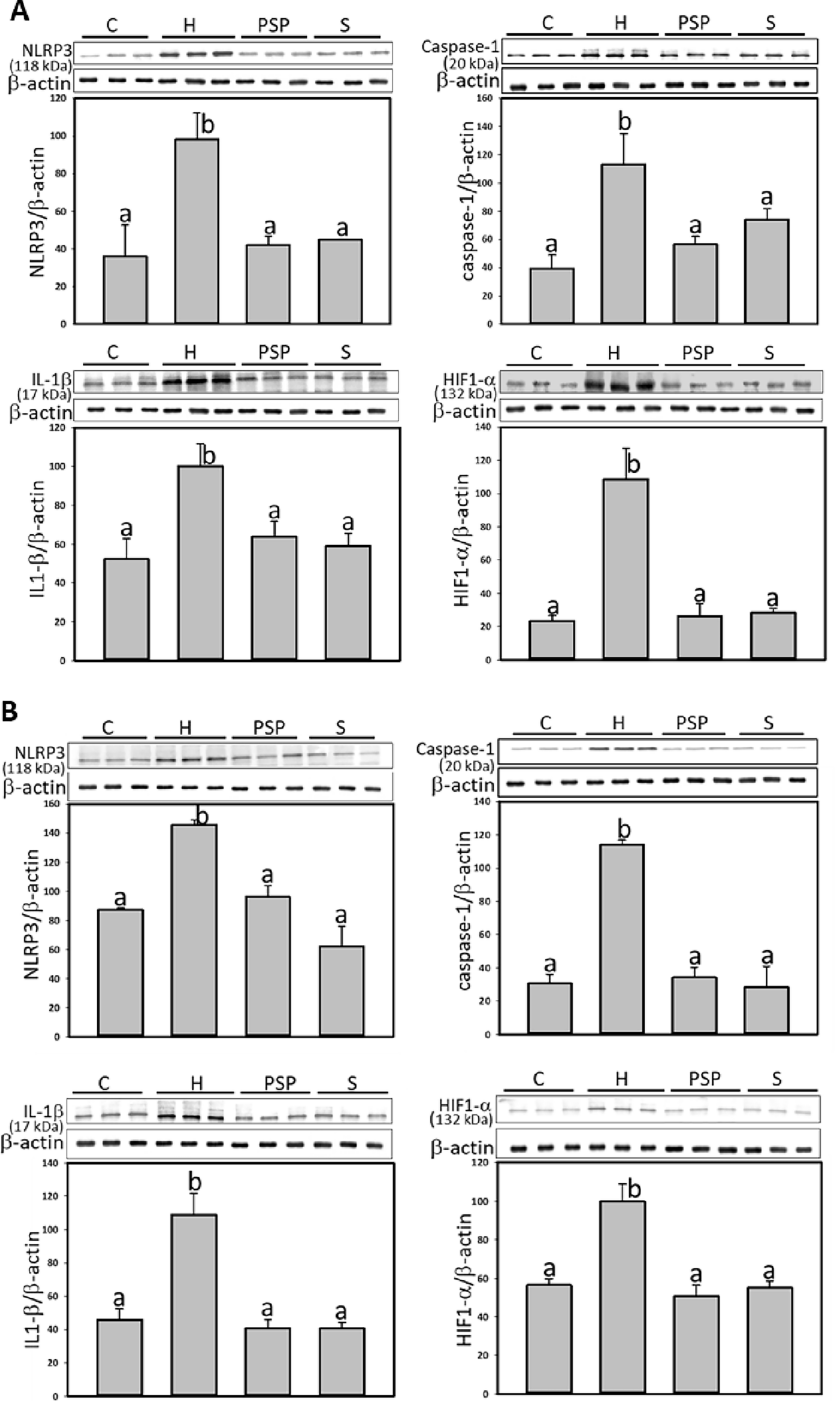
PSP reduced inflammasome-associated proteins in adipose tissue of HFD rats. A, subcutaneous (inguinal); B, visceral (mesenteric) adipose. β-actin was used as loading control. The relative image density was quantified by the densitometer. Values (mean ± SD) not sharing a common letter in the same row are significantly different (*p* < 0.05).

Obesity is associated with increased secretion of pro-inflammatory cytokines, thus we evaluated the expression levels of the inflammatory markers IL-6, TNF-α, and MCP-1 in both subcutaneous and visceral fat tissues. The HFD group exhibited elevated expression levels of these inflammatory proteins in both fat types. Conversely, in subcutaneous adipose tissues, significant reductions in TNF-α, IL-6, and MCP-1 were observed in the PSP group by 109.0, 104.1, and 110.1%, respectively; S group by 98.8, 124.3, and 113.4%, respectively, compared to H group (Figures 3). In visceral adipose tissues, significant reductions in TNF-α, IL-6, and MCP-1 were observed in the PSP group by 81.4, 178.3, and 65.4%, respectively; S group by 102.7, 138.4, and 74.2%, respectively, compared to H group. We also assessed the expression of inflammasome-associated proteins, including NLRP3, Caspase-1, IL-1β, and HIF-1α. The HFD group showed significantly increased expression of these proteins in both subcutaneous and visceral fat compared to the control group. However, in subcutaneous adipose tissues, PSP treatments significantly decreased the expression of NLRP3, cleaved Caspase-1, IL-1β, and HIF-1α by 90.3, 76.9, 75.8, 97.0%, respectively; atorvastatin treatments significantly decreased the expression of NLRP3, cleaved Caspase-1, IL-1β, and HIF-1α by 85.9, 53.2, 85.5, 94.4%, respectively, compared to H group. In visceral adipose tissues, PSP treatments significantly decreased the expression of NLRP3, cleaved Caspase-1, IL-1β, and HIF-1α by 84.9, 95.7, 108.4, 113.0%, respectively; atorvastatin treatments significantly decreased the expression of NLRP3, cleaved Caspase-1, IL-1β, and HIF-1α by 142.9, 102.4, 108.6, 103.2%, respectively, compared to H group (Figures 4).

### 3.6 PSP Promotes Browning of White Adipose Tissue

**Figure 5:**
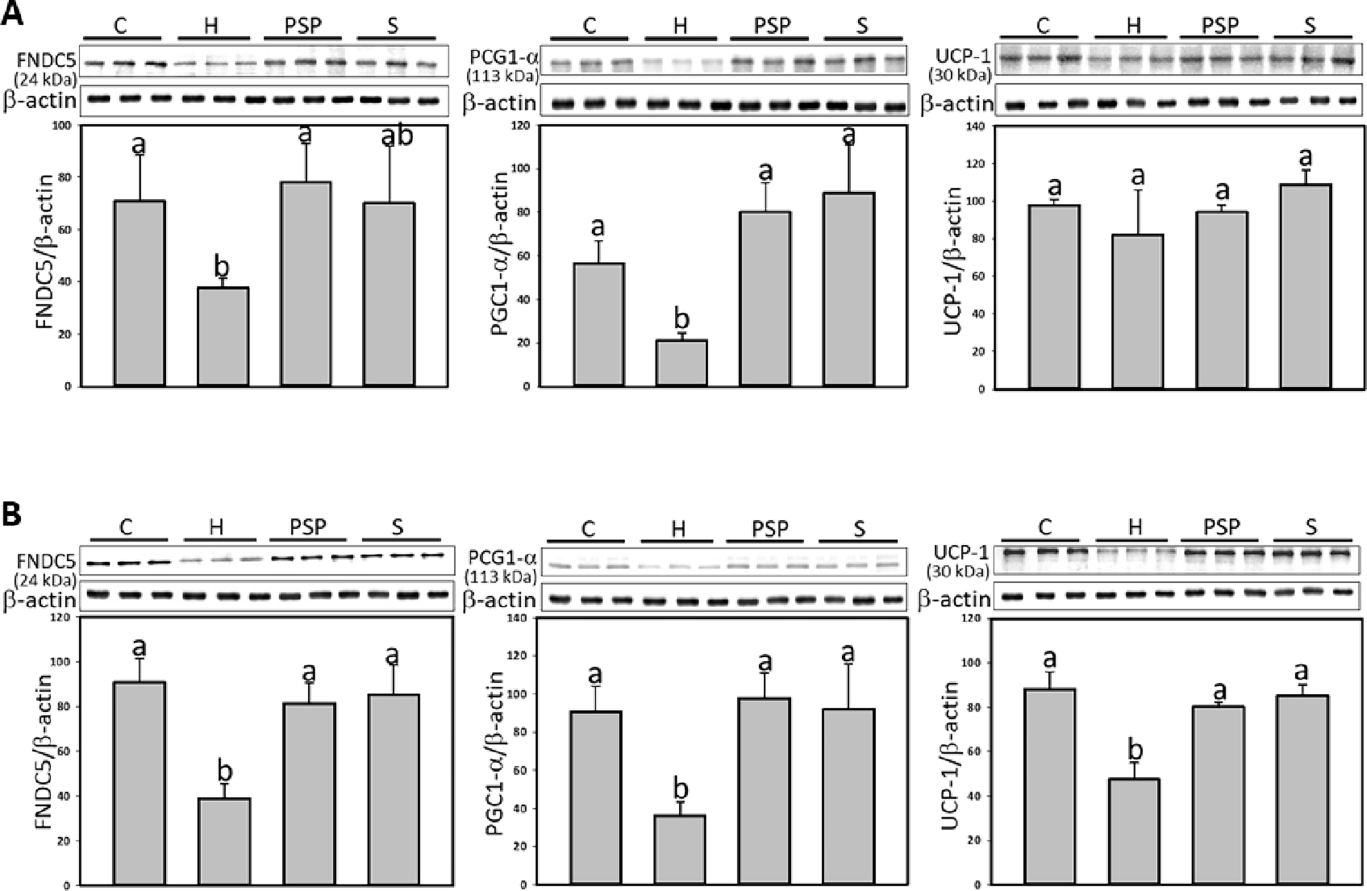
PSP enhanced the browning of adipose tissue of HFD rats. A, subcutaneous (inguinal); B, visceral (mesenteric) adipose. β-actin was used as loading control. The relative image density was quantified by the densitometer. Values (mean ± SD) not sharing a common letter in the same row are significantly different (*p* < 0.05).

The browning of white adipose tissue, a process of forming beige adipose tissue regulated by proteins such as FNDC5, PGC-1α, and UCP-1, was assessed. As shown in Figures 5, FNDC5 and PGC-1α were reduced in subcutaneous adipose tissues in the HFD group. As compared to HFD group, PSP treatment resulted in increased PGC-1α expression by 167.8%, leading to elevated FNDC5 protein levels by 122.4%. In visceral adipose tissue, all the proteins were significantly reduced in HFD group. PSP treatments significantly increased the expression of FNDC5, PGC-1α, and UCP-1 by 82.1, 113.0, 80.7%, respectively; atorvastatin treatments significantly increased the expression of FNDC5, PGC-1α, and UCP-1 by 89.5, 102.6, 92.5%, respectively, compared to H group.

## 4 Discussion

In the current study, rats received 2-week HFD to increase the body weight subsequently PSP supplement was administered to investigate the effects on body fat regulation, including lipid metabolism, inflammation, and adipose tissue browning. The results provide compelling evidence that PSP has a significant impact on body weight reduction, fat accumulation, and the modulation of molecular pathways associated with adipogenesis, inflammation, and browning of white adipose tissue.

Despite similar energy intake across all groups, the PSP-supplemented group exhibited a significant 76.5% decrease in body weight compared to the HFD group, highlighting the potential of PSP in mitigating obesity. This effect was more pronounced than that observed with atorvastatin, a known lipid-lowering agent, which reduced body weight by 55.2%. An anthocyanin-rich extract from *Euterpe oleacera* Mart. supplemented in HFD-fed male C57BL/6J mice for 14 weeks has been shown to gain less body weight.^25^ Anthocyanins extracted from a *Lycium ruthenicum* fruit fed to HFD-treated mice also has a reduced body weight.^26^ A daily anthocyanin intake of approximate 11.6 mg/day is recommended for individuals over 20 years of age in the USA population.^27^ Herein, 100 g of PSP powder containing approximately 8.4 mg of anthocyanins is capable to calculate the daily consumption. Additionally, a study indicated that the fiber of PSP is ranged at 4.07–5.11%, the soluble and insoluble fiber content between 1.20 and 1.63 and 13.53-21.91% respectively.^28^ Dietary fiber is well-established for its roles in satiety and lipid absorption. However, our current study did not detect significant differences in food intake between the HFD and PSP groups. These findings suggest that the body weight-reducing effects of PSP group may be partially attributed to the inhibitory effects of dietary fiber on lipid absorption. Further investigation is necessary to elucidate this mechanism.

Interestingly, PSP significantly reduced visceral fat in the obese animals, particularly in the mesenteric and perirenal regions in the current study. Lee et al. treated mice with a high-fat diet and PSP subjected to fermentation and alcohol extraction (100 mg/kg/day) for 4 weeks to reduce the weight of retroperitoneal white adipose tissue, epididymal white adipose tissue and liver accumulation of lipids.^23^ Rather than inducing obesity in animal models, Lee et al. concurrently administered PSP extract and HFD to assess the efficacy of PSP extract during the development of obesity.^23^ In our study, following the induction of weight gain, HFD-fed rats were supplemented with PSP powder at a dose of 100 mg/kg/day for 19 weeks to evaluate the long-term effects of nonextracted PSP on body weight and fat mass. This approach more closely mimics the real-world scenario of obese individuals using dietary supplements. While fermented and extracted PSP has demonstrated efficacy in reducing body fat, the extraction process may compromise the retention of certain bioactive components and limit our understanding of its long-term physiological effects. The use of nonextracted PSP in our study aligns more closely with dietary consumption patterns. In the mechanism of fat accumulation, diet enriched in high fat is associated with abnormal fat metabolism, primarily due to excessive caloric intake, leading to increased fat accumulation within the body. This surplus fat is stored in adipose tissue, resulting in abnormal lipid accumulation. The expansion of adipocytes in the adipose tissue subsequently triggers chronic inflammation, which has been implicated in the pathogenesis of metabolic syndrome-related diseases.^29,30^ The ability of PSP to reduce visceral adiposity, especially compared to the subcutaneous fat depot, underscores its potential to target the fat depots most linked to metabolic dysfunction.

PSP supplementation significantly reduced total cholesterol levels, although no significant changes were observed in triglyceride, HDL-C, or LDL-C levels compared to the HFD group. This partial improvement in lipid profiles suggests that while PSP may not affect all aspects of lipid metabolism, it can still reduce overall cholesterol levels, potentially contributing to its antiobesity effects. Notably, the lack of significant changes in glucose levels suggests that the metabolic effects of PSP may be more pronounced in lipid regulation rather than glucose homeostasis. In this experiment, food intake and serum triglyceride levels were lower in the HFD group compared to the control group. Huang et al. (2004) suggested that reduced triglyceride levels in the HFD group might be due to increased triglyceride oxidation, leading to the formation of ketone bodies and carbon dioxide, which in turn can suppress appetite and decrease food intake.^31^ Huang also indicated that rats fed with high-fat and high-fructose diet for 8 weeks show high serum triglyceride than that in HFD-fed rats, while rats fed HFD present a higher amount of adipose tissue than that in high-fructose-fed rats.^31^ It reveals that HFD may exert divergent effects on metabolism and regulating adipose tissue in rats. In the present study, the HFD group and the HFD-fed PSP or atorvastatin group exhibited significantly lower triglyceride levels compared to the control group. Moreover, serum ketone body levels were significantly elevated in the HFD, PSP, and atorvastatin groups (2.49 ± 0.38, 2.67 ± 0.24, and 2.37 ± 0.40 mg/dL, respectively) compared to the control group (1.95 ± 0.20 mg/dL)(data not shown in Results). Elevated ketone bodies may have contributed to reduced food intake in the HFD, PSP, and atorvastatin groups. Although the impact of PSP and atorvastatin supplementation on blood lipid profiles remains inconclusive, a high-fat and high-fructose diet may provide a more robust model for inducing obesity in future studies.

Histological analysis revealed that PSP significantly reduced adipocyte size in both visceral and subcutaneous adipose tissues, with a more marked effect on visceral fat. This reduction in adipocyte size aligns with the decreases in body fat, particularly in mesenteric fat, a key visceral fat depot. These findings suggest that PSP not only reduces the overall mass of fat tissue but also influences the cellular mechanisms underlying adipocyte hypertrophy or hyperplasia. Adipose tissue expansion is a complex process governed by the interplay between hyperplasia and hypertrophy, primarily regulated by a combination of genetic factors and excess energy intake.^32^ PPARγ is predominantly expressed in adipocytes and is a key regulator of adipocyte differentiation and fat storage, inhibition of this pathway suggests potential therapeutic implications for obesity management.^33^ Anthrocyanin extracts or delphinidin-3-O-β-glucoside have been reported to reduce the expression of PPARγ, FAS, and ACC1, indicating their role in modulating lipogenesis pathways and potentially reducing obesity.^33^ Kongthitilerd et al. demonstrated that culturing 3T3-L1 preadipocytes with 50 μM cyanidin for 4 days significantly reduced the expression of adipogenic genes PPARγ, C/EBPα, and aP2 (fatty acid-binding protein).^34^ The inhibition of FAS, ACC1, and PPARγ points to a suppression of de novo lipogenesis, which could account for the decrease in fat accumulation and adipocyte size. PSP with anthocyanins affecting lipid metabolism appears to target both visceral and subcutaneous fat, with a stronger impact on visceral depots, which is consistent with its preferential reduction of visceral fat mass. Regarding the effect of anthocyanin on reducing fat synthesis, previous studies have mostly used cell models for verification. In this current study, we cannot yet determine whether anthocyanin in PSP is the main factor in reducing body fat. In future studies, we will analyze the types of anthocyanin compounds in PSP and treat animals with a dose of anthocyanin equivalent to 5% PSP to compare the results with the current findings, then to confirm the role of anthocyanin in reducing body fat in HFD-fed rats and in regulating adipocyte hyperplasia and/or hypertrophy.

Adipose tissue functions as an active endocrine and immune organ, regulating inflammation through the production of fatty acid-binding proteins, adipokines, leptin, and lipid droplet-associated proteins. Obesity is often accompanied by chronic inflammation, with adipose tissue secreting various pro-inflammatory factors such as MCP-1, TNF-α, IL-1β, and IL-6. MCP-1 secretion recruits C–C chemokine receptor 2 to adipose tissue, facilitating monocyte accumulation and differentiation into macrophages, which can shift from an anti-inflammatory M2 phenotype to a pro-inflammatory M1 phenotype in obese adipose tissue.^30,35,36^ In our study, the results demonstrate that PSP exerts potent anti-inflammatory effects, as indicated by the significant reductions in pro-inflammatory cytokines, including TNF-α, IL-6, and MCP-1, in both subcutaneous and visceral adipose tissues. These cytokines are known to contribute to chronic low-grade inflammation, a hallmark of obesity, and are associated with insulin resistance and other metabolic disturbances. PSP with anthocyanins is capable to downregulate these inflammatory markers suggests that it can ameliorate obesity-related inflammation, which may further enhance its protective effects against metabolic dysfunction.^37,38^

Previous studies have highlighted the effect of PSP pigments in mitigating obesity and liver damage in HFD-fed mice. PSP pigments has been shown to suppress NLRP3 inflammasome activation and reduce inflammation-related protein expression in the liver.^39^ A clinical trial also demonstrated that anthocyanin supplementation significantly reduced NLRP3 inflammasome-related mRNA levels and serum concentrations of IL-1β and IL-18 in patients with nonalcoholic fatty liver disease (NAFLD).^40^ Adipose tissue macrophages are key players in adipose tissue inflammation, responding to microenvironmental cues and modulating adipose tissue remodeling and metabolic processes in a context-dependent manner.^41^ Zhang et al. demonstrated that the macrophages contribute to metabolic inflammation by increasing IL-1β production in adipose tissue, and ApoE can modulate the priming and activation steps of the NLRP3 inflammasome to reduce adipose tissue hyperstrophy.^41^ In the current study, the suppression of inflammasome-related proteins, such as NLRP3, Caspase-1, IL-1β, and HIF-1α, further supports the anti-inflammatory properties of PSP. The inhibition of these proteins, particularly in visceral adipose tissue, suggests that PSP may help mitigate inflammation-driven adipose tissue dysfunction, a key factor in the pathogenesis of obesity-related metabolic disorders.

One of the most intriguing findings of this study is the ability of PSP to promote the browning of white adipose tissue, as evidenced by increased expression of browning markers such as FNDC5, PGC-1α, and UCP-1. Browning is a process by which white adipocytes acquire characteristics of brown adipocytes, including enhanced thermogenesis and energy expenditure. PSP significantly upregulated these markers in both subcutaneous and visceral fat, particularly in the latter, suggesting that PSP may induce a thermogenic program that enhances energy expenditure and reduces visceral fat accumulation. Previous research has shown that citrus fruit consumption, rich in flavonoids, can increase the expression of irisin, PGC-1α, and UCP-1, thereby enhancing thermogenesis and promoting weight control.^42^ Lee et al. reported that PSP extract administration in mice increased the expression of genes related to the browning of inguinal white adipose tissue, such as PGC-1α and UCP-1. The treatment of HFD with PSP enhanced energy expenditure and exhibited the prevention of HFD-induced metabolic disorders.^23^ Our study corroborated these findings in treating the obese animals, as PSP supplementation significantly increased FNDC5 and PGC-1α protein expression in both subcutaneous and visceral fat, with a marked increase in UCP-1 expression in visceral fat. The promotion of adipose tissue browning is particularly important in the context of obesity, as it could shift the energy balance toward increased energy expenditure and fat burning. This mechanism, in conjunction with reduced lipogenesis and inflammation, positions PSP as a promising dietary intervention for the treatment of obesity and its metabolic complications.

Zheng et al. investigated the effects of atorvastatin on hyperlipidemic rats by administering low-dose (5 mg/kg/day) and high-dose (20 mg/kg/day) treatments for 4 weeks. The high-dose atorvastatin group exhibited significant reductions in total cholesterol, triglycerides, and LDL-C, while the low-dose group did not show significant changes.^43^ Similarly, in our study, administering atorvastatin to HFD rats at a dose of 10 mg/kg three times per week did not result in significant alterations in total cholesterol, triglycerides, or LDL-C levels. Although both PSP and atorvastatin had beneficial effects on body weight, fat mass, and inflammation, PSP appeared to exert a more pronounced effect on adipose tissue browning and lipogenic pathways. Atorvastatin is known primarily for its lipid-lowering effects, but the broader impact of PSP on inflammation and adipocyte metabolism suggests that it may offer additional benefits beyond lipid regulation. This multifaceted mechanism of action highlights PSP potentiating as a complementary or alternative therapy to traditional pharmacological treatments for obesity.

The findings of this study suggest that PSP may hold potential as a dietary intervention for obesity; however, some limitations are needed to consider when interpreting the results. First, this study utilized HFD-induced obesity model in rats. Although rodent models are commonly used in metabolic research, the physiological responses observed in rats may not fully replicate those of humans. Therefore, it should be considered when extrapolating these findings to human obesity and metabolic disorders. Second, the 19-week duration of PSP supplementation may not sufficiently capture the long-term effects on body fat regulation and metabolic health. Given that obesity is a chronic condition, longer-duration studies are needed. Third, in line with a prior study, the present study employed a single concentration of PSP (5% supplementation) in the HFD. It remains uncertain whether PSP elicits dose-dependent effects on body fat, lipid metabolism, and inflammation. Future studies are considered to investigate a range of PSP dosages to determine the optimal level for obesity treatment and prevention. Fourth, PSP, being rich in fiber and bioactive compounds, may modulate the gut microbiome, contributing to the observed metabolic effects. Characterizing changes in gut microbiota composition could provide insights into the mechanisms through which PSP influences body fat accumulation and systemic inflammation. Lastly, while atorvastatin was included as a comparator for lipid-lowering effects, additional antiobesity drugs that specifically target weight loss or fat distribution should be considered in future research to broaden the understanding of how PSP compares with current therapies in terms of efficacy and safety. Addressing these limitations will allow for a more comprehensive understanding of the role of PSP in obesity management and its potential application in human health.

In summary, PSP supplementation significantly reduces body weight, visceral fat accumulation, and adipocyte size while modulating key molecular pathways involved in lipogenesis, inflammation, and adipose tissue browning. These findings suggest that PSP has the potential to be a valuable dietary intervention for the prevention and treatment of obesity and its related metabolic disorders. Further studies are warranted to elucidate the long-term effects of PSP and its applicability in human populations.

## Data Availability

The data used to support the findings of the study are available upon reasonable request from the corresponding author.

## Supporting Information Available

Change of body weight in HFD-fed rats (Table S1); weight of food intake in HFD-fed rats (Table S2); organ weight of heart, liver, spleen, and kidney in HFD-fed rats (Table S3) (PDF)
