# Supplementary material for: Purple Sweet Potato Ameliorates High-Fat Diet-Induced Visceral Adiposity by Attenuating Inflammation and Promoting Adipocyte Browning
Source: J Agric Food Chem. 2025 Feb 3;73(6):3457–67. doi: 10.1021/acs.jafc.4c08799 (PMC11826983; doi:10.1021/acs.jafc.4c08799)
Supplement: Supplementary file 1 — jf4c08799_si_001.pdf [file jf4c08799_si_001.pdf]

Purple Sweet Potato Ameliorates High-Fat Diet-Induced Visceral Adiposity by Attenuating Inflammation and Promoting Adipocyte Browning

Chi-Hua Yen, Ming-Hui Chiang, Yi-Chen Lee, Erl-Shyh Kao, Huei-Jane Lee

Electronic Supporting Information

Table of contents

Table S1. The change of body weight in HFD-fed rats

Table S2. The weight of food intake in HFD-fed rats

Table S3. The organ weight of heart, liver, spleen and kidney in HFD-fed rats

Table S1. The change of body weight in HFD-fed rats.

Rats were separated as a control group on a normal diet, AIN-93M, Bio-Serv, Flemington, NJ, USA, 3.58 kcal/g, 4.1% calories from fat; HFD group, AIN-93M diet supplemented with lard, 4.35 kcal/g, 43% calories from fat; PSP group, HFD mixed with 5% (w/w) purple sweet potato powder, fed every day; S (statin) group, HFD+atorvastatin group, HFD with atorvastatin, 10 mg/kg body weight administered via gavage 3 times per week. The body weight shown in Table 1S below was recorded daily and averaged weekly. The body weight results were described in the main text (Material 2.2 and Table 1).

Body weight (g)	Control	HFD	PSP	S
0 week	249.02±1.41 ^{a}	250.61±2.80 ^{a}	248.43±1.74 ^{a}	250.28±0.90 ^{a}
1st week	281.91±10.53 ^{a}	317.12±9.65 ^{b}	295.88±10.55 ^{a}	319.16±5.33 ^{a}
2nd week	322.36±14.77 ^{a}	362.27±21.52 ^{b}	361.47±21.66^{b}	362.94±20.54^{b}
3rd week	371.30±18.84 ^{a}	416.47±26.68 ^{b}	407.32±28.02 ^{b}	414.51±26.43 ^{b}
4th week	376.17±15.82 ^{a}	444.17±28.41 ^{b}	422.37±25.10 ^{b}	439.39±27.75 ^{b}
5th week	420.67±16.53 ^{a}	488.11±35.01 ^{b}	447.20±24.13 ^{b}	480.63±32.35 ^{b}
6th week	449.74±15.38 ^{a}	516.54±36.67 ^{b}	467.00±21.23 ^{a}	494.53±34.74 ^{b}
7th week	455.12±14.75 ^{a}	534.38±38.09 ^{b}	483.46±18.00 ^{a}	519.95±32.40 ^{b}
8th week	463.49±19.11 ^{a}	546.48±42.49 ^{b}	492.67±18.63 ^{a}	533.68±33.23 ^{b}
9th week	471.86±24.73 ^{a}	558.59±47.87 ^{b}	501.88±20.36 ^{a}	547.41±34.82 ^{b}
10th week	493.34±21.47 ^{a}	586.52±53.05 ^{b}	519.99±25.95 ^{a}	574.48±38.54 ^{b}
11th week	515.22±23.55 ^{a}	608.33±57.07 ^{b}	541.69±24.03 ^{a}	595.04±39.90 ^{b}
12th week	540.89±28.73 ^{a}	627.33±61.25 ^{b}	562.89±29.80 ^{a}	611.75±44.62 ^{b}
13th week	550.67±26.95 ^{a}	639.11±63.13 ^{b}	584.11±30.32 ^{a}	626.50±46.37 ^{b}
14th week	560.11±9.11 ^{a}	639.56±55.98 ^{b}	594.44±30.76 ^{a}	632.38±43.59 ^{b}
15th week	568.56±29.88 ^{a}	657.22±59.22 ^{b}	601.44±36.58 ^{a}	639.50±42.49 ^{b}
16th week	556.11±34.03 ^{a}	646.44±61.25 ^{b}	583.33±28.57 ^{a}	617.13±42.28 ^{bc}
17th week	561.78±35.34 ^{a}	658.22±62.67 ^{b}	578.67±31.85 ^{a}	604.88±42.28 ^{a}
18th week	557.89±40.64 ^{a}	680.44±61.19 ^{b}	564.67±34.06 ^{a}	600.63±45.32 ^{a}
19th week	549.23±35.18 ^{a}	682.33±48.80 ^{b}	573.44±32.31	615.88±36.45 ^{c}
20th week	550.44±37.98 ^{a}	681.25±56.87 ^{b}	580.34±35.39 ^{a}	609.72±38.78 ^{c}
21st week	555.23 ± 26.39 ^{a}	686.02±47.04 ^{b}	585.76±25.87 ^{ac}	613.82±16.79 ^{c}

Table S1. The change of body weight in HFD-fed rats. The rat fed HFD was 686.02±47.04 gram in final body weight that showed significant increase compared to the control group ($p < 0.05$), whereas the groups of PSP and S were shown significant reduction, respectively, compared to HFD group. Values (means ± SD, n = 5) not sharing a common letter in the same row are significantly different ($p < 0.05$).

Table S2. The weight of food intake in HFD-fed rats

Rat grouping and treatment were described in section 1 above. The weight of food intake shown in Table 2S below was recorded daily, adjusted weekly by the body weight change, and averaged monthly. The results of food intake weight were described in the main text (Table 1).

**Food intake (g)**	Control	HFD	PSP	S
1st month	29.11	22.9	23.79	22.69
2nd month	28.92	20.28	20.05	22.08
3rd month	29.05	19.55	20.04	19.65
4th month	23.99	20.24	19.89	19.47
5th month	27.2	20.29	20.13	19.53
Average	27.7 ± 2.2 [a]	20.7 ± 1.3 [b]	20.8 ± 1.7 [b]	20.7 ± 1.6 [b]

Table S2. The weight of food intake in HFD-fed rats. The results showed that the average weight of food intake was lower in HFD group compared with the control. The food intake weight among HFD, PSP, and S group was not different. Values (means ± SD, n = 5) not sharing a common letter in the same row are significantly different ($p < 0.05$).

Table S3. The organ weight of heart, liver, spleen, and kidney in HFD-fed rats

After the animal was euthanized, the organs of the heart, liver, spleen, and kidney were weighed and shown in Table 3S below.

Organ weight (g)	Control	HFD	PSP	S
Heart	1.57 ± 0.05	1.63 ± 0.07	1.65 ± 0.05	1.74 ± 0.08
Liver	13.49 ± 0.34	13.93 ± 0.62	12.71 ± 0.30	12.79 ± 0.34
Spleen	0.76 ± 0.03	0.72 ± 0.03	0.64 ± 0.02	0.71 ± 0.030
Kidney	3.57 ± 0.13^{a}	3.81 ± 0.11^{b}	3.42 ± 0.05^{a}	3.82 ± 0.11^{b}

Table S3. The organ weight of heart, liver, spleen and kidney in HFD-fed rats. The results showed that the average weight of heart, liver, and spleen was not different among the groups of control, HFD, PSP, and S. The kidney weight in HFD group was significantly increase compared with that in control, while PSP treatment reduce the kidney weight significantly compared with HFD group. Values (means ± SD, n = 5) not sharing a common letter in the same row are significantly different ($p < 0.05$).
